# The Organic Diseases of the Bones

**Published:** 1841-07

**Authors:** 


### ART. X.

*Die Organischen Knochen-Krankheiten.* Ein Lehrbuch von Dr. A. L. RICHTER.—*Berlin,* 1839. 8vo, pp. 208.
*The Organic Diseases of the Bones.* A Manual by Dr. A. L. RICHTER.—*Berlin,* 1839.

THE volume whose title is prefixed to the present article is the production of an esteemed and experienced surgeon of Germany, and constitutes a very fair summary or compendium of the present state of our knowledge in the department of surgery to which it relates. The whole subject of organic affections of the osseous system is examined and discussed in a candid and judicious strain; and although the author obtains his materials chiefly from the labours of others, he is yet manifestly not to be regarded as a mere compiler. We do not, it is true, rise from the perusal of the work with any conviction that the author has himself added much to what was before ascertained, with respect to the class of diseases therein discussed. We do not, however, think that on this account its utility is much diminished. It is highly meritorious to have industriously sought out the published results of others, and to have candidly examined the amount of their value and practical importance. In this view of the case, we regard the construction, in a well-arranged and readable style, of a manual like the present, embracing all that is of moment upon any given subject, as a decidedly *useful* undertaking. On this account then, but more particularly as a large class of practitioners, most of those indeed unconnected with large hospitals, obtain from their own observation but little acquaintance with diseases of the bones, and this little only from the treatment of isolated cases, we think that a few of our pages will not be ill employed in furnishing a brief analysis of some of the more interesting matter contained in the volume before us.

The principle upon which our author rests, and from which he never swerves throughout the work, consists in the recognition of a pathological analogy between diseases of the bones and those of the other textures. That our knowledge of the former did not for a long time, maintain equal pace with that of the latter was owing, he states, to the circumstance of its being the custom to regard bone rather as a mere aggregation of lifeless matter, than as living structure, participating in the general properties of the organization. In this state of things, any parallelism between disease occurring in bone and that developing itself elsewhere could hardly be attempted. The older writers would include the most dissimilar pathological conditions, in the employment of the same terms; and the expressions "caries," "exostosis," and "spina ventosa," were used in the most vague and arbitrary manner; a proceeding, of course, which gave rise to a treatment equally irrational and empirical. Petit, whose work appeared in 1705, was the first to institute a comparison between disease occurring in bone and that taking place in the soft tissues; and, although he made no new classification of the forms of disease, a foundation was yet laid for a future scientific advance in this department. The highly interesting researches of Haller, Duhamel, Detlef, Troja, Köler, and others, on the physiology of bone could not but exert a favorable influence upon the labours of those engaged in the study of its diseases; although a long time elapsed before a very general application of such researches was made to the consideration of morbid alterations. The appearance of Boyer's work, at the commencement of the present century, marked another stage in the progress of knowledge in this branch of surgery; and it for some time remained the principal source whence other writers drew their materials. Within the last twenty years, however, a much more comprehensive acquaintance with the subject has been obtained, chiefly through the exertions of Scarpa, Béclard, Müller, Cruveilhier, Dupuytren, and others; and at the present day it is very generally allowed that the osseous structure may undergo just the same morbid changes as the rest of the tissues, and that any peculiarities seeming to exist therein are to be regarded not as something without parallel in the soft parts, but as modifications dependent upon the low measure of vitality present in bone. This, as we have before stated, is the fundamental principle with which Dr. Richter sets out, and the one which he steadily maintains in the progress of the work.

The whole matter is brought before the reader in twenty-one brief chapters, the pathological classification being for the most part such as we approve. In each division, the morbid alteration examined is regarded under five heads: the general characters of a disease being first given, then the diagnosis, next its etiology, then the prognosis, and lastly the treatment. The whole is then wound up by a copious bibliographical reference. Our author, after introducing the subject generally, enters, in the first chapter, into the discussion of inflammation of the bony structure, following, in this respect, the common example of pathological writers, in awarding the first place to this important lesion of the organization.

*Inflammation* of the osseous structure most commonly originates in the medullary and spongy texture, owing to its higher vitality, gradually involving, however, the denser lamellated tissue. The rise and progress of an attack is perfectly analogous to what obtains in the corresponding affection of other symptoms. Accordingly, inflammation of bone admits of the customary division into acute and chronic. In its general consequences, the latter is the more serious form, owing to its being nearly always more or less associated with some extensive constitutional derangement. The bony tissue in children and young persons, possessing a richer endowment of nutrient blood-vessels than what occurs in more advanced life, is thereby rendered at this period more liable to the affection. The symptoms are such as ordinarily characterize inflammation in other parts, modified only by the lower vitality of the involved structure. It is to be remarked, however, that after an attack has fairly established itself, the pain is at times to be recognized rather in the neighbouring joint than in the immediate seat of the evil. The causes of the affection may exist in the habit of body, or they may be formed by some lesion from without. Syphilis, scrofula, gout, or rheumatism being present in the system, predispose greatly to an attack. Owing to the extent of the constitutional derangement in many cases, and to the difficulty of acting by treatment immediately upon the affected structure, the prognosis is often unfavorable. A successful issue is to be sought entirely from the employment of measures rationally calculated on the one hand to subdue local excitement, and on the other to improve the general health. All vaunted specifics, in vogue with our fathers, must be cautiously avoided. In a short chapter upon Periostitis, we are told that it seldom or never presents an acute character, except when the causes have been either mechanical violence, some metastasis, or bad treatment of the chronic form.

Suppuration may arise in the osseous structure as a consequence of inflammation, and generally under the same conditions that mark its development in other parts. It is often confounded, but erroneously, with caries, or ulceration of the bony tissue; the processes, however, are just as distinct in the present instance as elsewhere, the one constituting essentially a formative effort, and the other being in its nature destructive. As in the soft parts, the two processes may coexist or may run the one into the other, but there is no identity. Abscesses in bone, when detected during life, have nearly always been found in the tibia, near the extremities. The signs of an invasion of this sort are, for a long time, somewhat obscure; swelling of the affected part comes slowly on, but little or no pain upon pressure is experienced until after some time has elapsed. The neighbouring joint, in the cases hitherto observed, has not participated in the morbid condition. Purulent matter in these instances often forms also beneath the periosteum, owing to its becoming involved in the attack. Sometimes there has been noticed after death a considerable deposit of bony matter, easily separated from the subjacent shaft, from the intervention of the periosteum between it and the adventitious formation. The locality of the purulent accumulation is primarily in the centre of the bone. It is an affection which authors have sometimes confounded with exostosis. It is also one of those to which the stupid and unmeaning term "spina ventosa" has been applied. Dr. Richter, in his account of the treatment of this morbid state, seems to expect little good from anything but amputation or the use of the trepan; and even where the case is encountered in its early stage, he does not think that much advantage is likely to be obtained from any attempt to subdue the affection by a milder proceeding; an opinion that may be true, but nevertheless, judging from analogy, one we think that should not be regarded as decisive of the point. As unquestionably a chronic inflammation or something analogous most commonly, if not invariably, precedes the formation of matter, we should always be disposed, in a suspected case of this kind, to attempt to subdue such inflammation by the means that are often found efficacious in corresponding affections of the other tissues; and this, in the expectation that, afterwards, the absorbents might remove any slight purulent deposit.

*Caries* is defined to be a process in bone analogous to ulceration in the soft parts, a definition, we are informed, only established in modern times. It has been stated, however, that such a comparison is as old as Galen; and certainly, we ourselves remember to have met with almost the very words of the definition in Duverney's treatise, published in the middle of the last century. It is an affection that occurs principally in the spongy structure of bone, although there is no portion of the osseous system which possesses an immunity. In all its character and relations, the analogy between this disease and ulceration in other tissues is maintained. It may form the primary affection, or it may constitute the issue of some other. The same complications and constitutional modifications that affect the presence of ulceration elsewhere obtain also in the present instance. Hence, we have phagedænic and fungous caries, syphilitic and scrofulous, and so on. The causes of ulceration of bone are also of a character generally like those which excite a similar affection in the soft parts. Any local injury capable of producing inflammation of bone may give rise to caries; or it may depend chiefly upon some great constitutional disorder, especially the state induced by the abuse of mercury in syphilis. When nature brings about a cure of this lesion, it is either by the occurrence of a healthful suppuration, or the complete death of the carious portion, which is afterwards got rid of by exfoliation. In attempting a cure by the aid of art, we must seek then to imitate nature in her proceeding, so far as is practicable. Dr. Richter, after recommending strict attention to the general health, seems, in our opinion, when dealing with the local affection, to attach too much value to topical stimulants and to the actual cautery as a means of converting the ulcerated into dead bone. For many reasons, which our space will not allow us to adduce, we much prefer the more general practice recommended by British surgeons, especially by Mr. Syme, the removal of the carious portions of bone by the knife, in those cases where any violent proceeding of the kind seems indicated. Much interesting matter is found in the sections treating of caries in the individual bones, but our limits do not allow us to go into its examination.

*Necrosis* of bone corresponds to gangrene of the soft parts, just as caries does to ulceration. It forms the lesion to which the term "caries sicca" was formerly applied in contradistinction to "caries humida," the proper caries. The great comparative frequency of gangrene in the osseous system obviously results from its possessing a much lower vital activity than that which is found in the other systems; and it is in consequence of this being more especially the case with the shafts of the long bones that they so frequently become the subject of extensive or even complete necrosis. In the event of destruction of any part of the bony tissue, the reproductive energy of all the surrounding parts becomes exceedingly active; and this furnishes the reason why in many forms of organic disease the supervention of actual necrosis becomes a beneficial occurrence; whilst, on the other hand, the development of caries near the seat of necrosis is a most unfavorable circumstance. The discussion of this division of the subject occupies a considerable space in the volume before us, and the death of the part, its exfoliation, and the regeneration (as forming the three successive stages of cases of this kind) are each in succession examined and discussed. As the precise process whereby the regeneration of the entire shaft of one of the cylindrical bones occasionally occurs has been differently explained by pathologists, we will give the author's account of the same in his own words:

"Total necrosis is followed by a productive inflammation in all the tissues surrounding the affected bone, including the undestroyed periosteum. A plastic gelatinous effusion takes place from these, and at the bony extremities that have escaped necrosis; within the effused mass vessels are developed; and hereupon the various stages of ossification are successively observed; for, after it has become harder and more cartilaginous, it gains ever more in density and firmness through the deposit of bony earth which occurs at innumerable points, and this deposit is often very considerable, even while the sequestrum remains in the limb. At the inner mouth of the fistulous communications, no deposit of bony matter takes place, and an opening remains which allows access to the sequestrum, and exhibits a *cloaca* which only later undergoes obliteration, to which the contraction of the newly-formed cylinder, after the removal of the dead portion, certainly contributes very much. It is difficult to determine whether or not such newly-formed bone be again furnished with medullary tissue." (p. 92.)

*Mollities ossium* results from a diminished proportion of calcareous matter in the bones, arising for the most part in childhood and in old age. Taking place in the early period of life, it is of the nature of an arrest, so to speak, of the proper development of the bony texture; and its occurrence in old age constitutes a kind of retrogression in the organization. In the former case, osseous matter is insufficiently deposited in the *matrices* of the structure; in the latter, it becomes removed by interstitial absorption. But little is known of the determining causes of this affection, whether it presents itself as rickets in childhood, or occur in more advanced life. A damp climate is commonly regarded as conducive to the disease. Dr. Richter states that on this account it is unusually prevalent in our own country, and that it has obtained in consequence the designation of the "English disease," a national distinction of which we were previously aware, but of the justice of which we very much doubt. Associated with this affection is always observed extensive disorder in all the functions concerned in assimilation. A deposit of lime is often noticed in the urine. The vertebral column is generally the first to give way so as to exhibit deformity. The rickets of childhood seldom cripples to the same extent as the corresponding affection in more advanced life. Nearly all the cases recorded of very extraordinary disfigurement have been of adults. The prognosis in children is often very favorable. An improvement in the digestive organs is usually followed by a hardening of the bones; the induced deformity, however, seldom evincing much improvement until the attainment of puberty. Measures calculated to improve the functions of organic life generally are alone conducive to a cure. The utility of specifics in this affection is quite exploded.

In this brief account, we have hitherto followed our author somewhat in detail through the better half of his work, as the topics handled have possessed more practical interest than those of which he treats in the sequel. In our remaining notice, we can do little more than allude to the particular lesions, in the examination of which Dr. Richter forms distinct chapters. These consist chiefly of certain pathological changes of structure, of whose causation and successful treatment but little that is satisfactory is understood. The review of them, however, is interesting as still further exhibiting the entire analogy, especially in disease, subsisting between the osseous and the other systems.

*Osteosclerosis*, or hardening of bone, is exactly the opposite of the last considered affection, and is owing to a predominance of the earthy matter. It may be regarded as the analogue of induration in other textures. *Osteoporosis*, or porosity of bone, is formed by a loosening of the tissue, and results not from any absorption of the mass, but purely from the presence of an expansive activity. *Osteopsathyrosis* is a term employed to designate fragility or brittleness of bone, a state induced by defective nutrition and often prevalent in old age, though no period of life is exempt from its occurrence. A sort of atrophy of the osseous texture marked by a diminution of volume, sometimes takes place and is technically designated *Osteonabrosis*. Of these various changes in the structure but little explanation is to be afforded; they form peculiar lesions of the nutritive function, like in many respects to what occurs in the soft parts, and as little, or indeed less, amenable to treatment.

*Morbid growths* upon the surface and within the structure of bone are not of unfrequent occurrence. These vary in their characters according to the state of the constitution with which they are associated, and upon which they often depend. Thus syphilis and gout often lay the foundation of such affections. They may present themselves with characters of a mild or of a malignant nature, just as obtains with such diseases in the soft parts. Simple tumours of bony matter sometimes arise on the surface, called exostoses; and sometimes special formations, such as medullary fungus, the fibrous or the fleshy tumour maintaining in all respects a very close resemblance, in their apparent origin and progress, to such affections in the rest of the system. The tuberculous diathesis, as also the disposition to melanosis, allows no immunity to the bony structure; and even morbid conditions, such as dropsy and the formation of cysts, prevail here at times as elsewhere. Indeed, the further our knowledge advances in the morbid anatomy of the osseous structure, the more complete seems the unity in the essential characters of all the components of the organization; since it is now pretty well made out that even those parts formerly regarded as little more than lifeless supports to the true organism themselves participate in all the general properties of the entire system.

We cannot dismiss the subject without again recording our approbation of the manner in which Dr. Richter has executed his performance. Throughout the volume the style is free and plain, the arrangement is judicious, and, as it seems to us, a due relation is maintained between the amount of facts stated and the extent of their explanations, a rare quality with writers, especially in dealing with subjects of practical medicine. Indeed, we are much disposed to regard this manual as the best of its kind, as it furnishes systematically, in a very brief compass, almost everything of interest or value that has been ascertained in the department. We think that any one having a taste for such pursuits, would render a service to our English medical literature by translating the work into our own language. It would be found, we are sure, a most useful hand-book for students; and we are equally certain, that there are few practitioners who by its perusal would not either add to their prior stock of information, or find that they had profitably renewed or refreshed that which they had previously obtained.

